# ‘No matter what time of day’: The value of joining Facebook groups supporting women's self‐management of gestational diabetes mellitus

**DOI:** 10.1111/hex.14082

**Published:** 2024-05-24

**Authors:** Sheila Pham, Kate Churruca, Louise A. Ellis, Jeffrey Braithwaite

**Affiliations:** ^1^ Faculty of Medicine and Health Sciences, Centre for Healthcare Resilience and Implementation Science, Australian Institute of Health Innovation Macquarie University Sydney New South Wales Australia

**Keywords:** gestational diabetes mellitus, Internet, peer group, self‐help groups, social networking, survey

## Abstract

**Background:**

Gestational diabetes mellitus (GDM) affects a significant and growing proportion of pregnant women each year. The condition entails additional monitoring, self‐management and healthcare use during pregnancy, and some women also join GDM support groups on Facebook. Little is known about the practices inside these groups, but examining them may elucidate support needs, women's experience of healthcare and improve overall outcomes. The aims of this study were to explore motivations for joining GDM Facebook groups and the perceived value and benefits of such spaces.

**Design:**

A cross‐sectional design using a web‐based survey collected data from two peer‐led GDM Facebook groups; relevant quantitative and qualitative data were extracted from open and closed questions, and analysed using descriptive statistics and content analysis.

**Results:**

A total of 340 women responded to the survey, with 306 (90%) tendering their motivations to join a GDM Facebook group. Their answers were classified into six categories: peer support; information and practical advice; lived experiences; community; a safe place to ask questions and being recommended. The most commonly reported benefits of membership were ‘reading about food ideas’ and ‘finding helpful information and tips’. Respondents reported finding their group strongly sympathetic, sincere, compassionate, heart‐felt, tolerant, sensitive, warm and supportive.

**Discussion and Conclusions:**

GDM Facebook groups are valuable for informational and emotional support, and the sharing and perusal of lived experiences; another key benefit for women is feeling belonging to a community. GDM Facebook groups provide women with access to more tailored and readily available support, filling gaps not addressed by healthcare providers.

**Patient Contribution:**

This study was led by a person with lived experience of GDM, and the survey was pilot tested with women who had also experienced GDM, which contributed to its development.

## 1 INTRODUCTION

Pregnancy is a time of great change in a woman's life, where additional health‐related information is generally required to navigate the experience, particularly when symptoms and unexpected problems arise. For many, the Internet has become a key source of pregnancy‐related information,^1^, ^2^ including social media platforms such as Facebook.^3^ Facebook provides useful support for pregnant women given its low cost of access and ready availability.^4^ Support groups on Facebook for pregnant women may be created and moderated by healthcare professionals including midwives,^5^ as well as by peers; the latter are especially widespread, and an underresearched area with little known about practices within and across such online groups.^2^, ^3^, ^6^

Gestational diabetes mellitus (GDM) is defined as any degree of hyperglycaemia first recognised during pregnancy.^7^ It is a condition which affects a significant and growing proportion of pregnant women around the world each year.^8^ In Australia, for example, the prevalence amongst pregnant women more than doubled in less than a decade, from 8.3% in 2014 compared with 17.9% in 2021‐2022.^9^, ^10^ Internationally, the incidence and health burden of GDM is on the rise with the pooled global standardised prevalence of GDM estimated to be 14.0%, with the lowest prevalence in the Middle East and North Africa (7.5%) and the highest in South East Asia (27.0%).^8^

Previous studies have highlighted barriers to women with GDM receiving care, including lack of social support, practical information, and communication.^11^ Given the need for additional treatment and monitoring, and the pressure of self‐management which can be time‐consuming and exhausting,^12^ it is not surprising that peer support groups focused on GDM proliferate on Facebook as women feel the need for greater support. To date, there is relatively little research on experiences inside GDM groups. A recent qualitative study found that one such group was valuable as a community of support, with members holding each other accountable to their regimes of self‐management.^13^ Our study examining the posts in GDM Facebook groups also affirmed the value of virtual peer support, and suggested that examining what information is being sought and shared by participants in GDM online communities can help healthcare providers reflect gaps in information delivered through formal healthcare.^14^ Looking to a comparable condition, diabetes mellitus, a scoping review of 47 studies on the use of diabetes online communities found a variety of psychosocial benefits and few reports of negative consequences, though it was noted that such communities may not be beneficial for all.^15^

Further examination of why women join these groups and the expectations they hold may thus be illuminating for healthcare providers, as understanding patient support needs is critical to improving care outcomes.^16^ As such, the aims of this study were to explore: (1) the motivations to join such groups and what is being sought; and (2) why these groups are valued and the perceived benefits of membership.

## 2 PARTICIPANTS, ETHICS AND METHODS

### 2.1 Survey

A national web‐based cross‐sectional survey collected data from two peer‐led closed Facebook groups focused on GDM, founded and run voluntarily by independent individuals drawn from the community. These groups were chosen as the two largest focused on GDM in Australia; at the time of data collection, the combined membership of the two groups was over 6500 members.

### 2.2 Participant recruitment

A Facebook post promoting the study and inviting women to participate was shared in both groups, once per month for exactly 3 months, from the end of 2018 to the start of 2019. The initial post advertising the survey explicitly referenced S. P.'s positionality as someone who had experienced GDM, along with a brief overview of the study and a link to the online survey and further information.

### 2.3 Study design

The survey collected data on a variety of demographic characteristics (e.g., age, language background, education, income) and family history of diabetes, in addition to questions on experiences of GDM, healthcare, and the group. Responses to the latter of these topics were used to address the present study's aims; this included an open‐ended question combined with a number of related close‐ended questions. The use of qualitative and quantitative data was chosen to support exploration, potential data triangulation and more rigorous discussion. The questions are described in further detail below (also see Supporting Information S1: Appendix 1 for the survey questions). It should be noted that in the survey, GDM was referred to as ‘gestational diabetes’ or ‘GD’ to be accessible and familiar to respondents.

We pilot tested the survey with a diverse sample of 12 to check for clarity before recruitment of participants formally commenced. Of the 12, 5 had experienced GDM and 2 had experienced pregnancy and childbirth. Feedback received from pilot testing led to minor modifications to the survey.

#### 2.3.1 Motivations to join

Data around motivation to join a Facebook group was gathered from the responses to the open‐ended question: ‘Can you explain exactly what led you to join a GD Facebook group?’

#### 2.3.2 Value and benefits of membership

Perceived value and benefits of membership were collected from subsequent close‐ended questions. First, from the checkbox question, ‘What do you like about being in a GD group?’ (e.g., ‘reading about food ideas’); and second, from a semantic differential scale, ‘During my interactions in a GD Facebook group, I felt the community to be…’, with eight‐items answered using a 5‐point scale (e.g., ‘not compassionate’ to ‘compassionate’). The scale was adapted from work by Nambisan,^17^ who developed their scale to explore perceived empathy in online health communities, based on the assumption that this is something which is felt when someone posts and is responded to by others.

### 2.4 Data analysis

Demographic data were analysed descriptively. A summative content analysis^18^ was undertaken on data from the open‐ended question, an approach to analysing qualitative data which starts with identifying and quantifying certain words/content to understand contextual use, before applying latent content analysis. To this end, the first author independently read and reread each response, and keywords were identified (e.g., support, experience) as the basis of categories, allowing for multiple categories to be present within a single response (i.e., categories were not mutually exclusive). All authors then compared and confirmed the identified categories and further interpretation. A descriptive analysis was applied to the responses of the check box question. For the semantic differential scale, a scale‐by‐scale analysis was used.^19^

## 3 RESULTS

### 3.1 Demographic characteristics

Across the two Facebook groups, there were 340 survey participants, after incomplete surveys were excluded. The American Association for Public Opinion Research^20^ defines partials as cases that respond to more than 50% of all applicable questions. However, we determined that completing about two‐thirds (66%) was more applicable as the criterion for inclusion in the present study, as this threshold encompassed most of the essential questions, including both closed‐ and open‐ended questions about GDM healthcare experience, Internet use and a number of questions about joining a GDM Facebook group.

First, one‐way analysis of variance and *χ* ^2^ analyses were used to test whether we were justified in pooling the data from the two Facebook groups, Group 1 (*n* = 254) and Group 2 (*n* = 86). There were no significant differences between the group in terms of age (*F*(1, 338) = 2.66, *p* = .10), language spoken at home (*χ* ^2^ = 0.74, *p* = .39), family history of diabetes (*χ* ^2^ = 0.35, *p* = .84) and level of education (*χ* ^2^ = 1.12, *p* = .89).

The demographic characteristics of the samples are provided in Table 1. The age of respondents ranged from 20 to 45 years old, with a median age of 32. The vast majority were born in Australia (288, 84.7%) and spoke English as their main language (311, 91.7%). Almost half reported a family history of diabetes (163, 47.9%) and just over half of the sample had completed at least one university degree (148, 50.2%).

**Table 1:** Demographics of the sample: Group 1 (*n* = 254), Group 2 (*n* = 86) and total (*N* = 340).

	Group 1	Group 2	Total
	*n* (%)	*n* (%)	*N* (%)
Age			
20–25	25 (9.8)	1 (1.2)	26 (7.6)
26–30	72 (28.3)	25 (29.1)	97 (28.5)
31–35	97 (38.2)	42 (48.8)	154 (45.3)
36–40	51 (20.0)	13 (15.1)	64 (18.8)
41–45	9 (3.6)	5 (5.8)	14 (4.1)
Country of birth			
Australia	213 (83.9)	75 (87.2)	288 (84.7)
Other	41 (16.1)	11 (12.8)	52 (15.3)
Main language spoken at home			
English	234 (92.1)	77 (89.5)	311 (91.5)
Other	19 (7.5)	9 (10.5)	28 (8.2)
Family history of diabetes			
Yes	123 (48.4)	40 (46.5)	163 (47.9)
No	120 (47.2)	41 (47.7)	161 (47.4)
Not sure	11 (4.3)	5 (5.8)	16 (4.7)
Education			
Less than year 12	14 (5.5)	7 (8.1)	21 (6.2)
Year 12 or equivalent	22 (8.7)	7 (8.1)	29 (8.5)
TAFE qualification, technical, trade certificate, diploma or equivalent	72 (28.3)	25 (29.1)	97 (28.5)
Bachelors degree	62 (24.4)	18 (20.9)	80 (23.5)
Postgraduate degree or higher	51 (20.1)	17 (19.8)	68 (20.0)

*Note*: Valid percent reported only.

Abbreviation: TAFE, technical and further education.

### 3.2 Motivations for joining

There were a total of 306 responses (90%) to the free‐text question on what led to joining the group. The following six categories emerged about members' motivations to join: 1—peer support; 2—information and practical advice; 3—lived experiences; 4—community; 5—a safe place to ask questions; 6—being recommended (see Table 2). There was only one response (ID027) (*n* = 1) which was not categorised as it lacked sufficient detail (i.e., ‘out of interest’).

**Table 2:** Categories and number of occurrences out of total response (*n* = 306).

Categories	No. of occurrences	%
Peer support	168	49.4
Information and practical advice	154	45.3
Lived experiences	56	16.5
Community	37	10.9
Being recommended and self‐referral	48	14.1
A safe place to ask questions	22	6.5

*Note*: Inter‐ and intracategory responses were not mutually exclusive.

#### 3.2.1 Peer support

Overwhelmingly, ‘support’ was the keyword that defined this category. In the raw data, *support* and its derivative words—*supported, supporting, supportive*—appeared 131 times. In comparison, *experience(s)* appeared 58 times, followed by *people*, appearing 54 times. The category was titled ‘Peer support’ (*n* = 168), with almost half of these occurrences explicitly stating the desire for support from peers (e.g., ID014: ‘for more support and advice from other women in the same situation’), while the rest implied this in the desire for support from a group of women also encountering GDM (e.g., ID155: ‘To have other women to talk to who understand and can share experiences’). Some elaborated by explaining support was lacking in ‘real life’ (e.g., ID019: ‘I needed support and none of my real friends had gone through GD’). Support was also referred to as being needed due to a deficit in formal healthcare (e.g., ID266: ‘Wanted a bit of extra support, as it seemed like ages between the initial diagnosis, then group session and the one on one dietician and educator’).

#### 3.2.2 Information and practical advice

The category of information and practical advice brought together responses which referred to seeking information in a generic sense (*n* = 44), as well as those which referred to specific information and advice, namely food ideas (*n* = 52), tips, tricks and advice (*n* = 40), diet (*n* = 9), exercise (*n* = 5) and birth (*n* = 2). According to the responses coded to this category, the information being sought by women was embodied and practical. As one woman (ID096) stated, ‘the hospital only gives you basic info’. Another respondent (ID112) wrote there was a ‘lack of information provided in my first pregnancy with GD so I searched on Facebook for a group and found one’, indicating a priori understanding that Facebook groups are repositories of knowledge and, in this case, able to redress a previously experienced information deficit about GDM.

#### 3.2.3 Lived experiences

A large number of respondents (*n* = 56) indicated they joined to read about ‘other people's experiences’ and share their own. This explicit motivation suggested that perusing ‘lived experiences’ was a distinct activity, though related to both information and support (e.g., ID062: ‘reading about others who have been on this journey already made me feel like I had support’). This type of intelligence was also seen by some as being more valuable than formal advice (e.g., ID248: ‘Experiences I have found are better than fact sheets which are very generic’). Another respondent (ID051) described how she was seeking ‘immediate feedback from women with firsthand experience who were also awake in the middle of the night’, pointing to how the online interactive group provided not just lived experiences but *live* experiences unfolding throughout all times of the day.

#### 3.2.4 Community

‘Community’, a separate category, was strongly expressed in the data (*n* = 37) (e.g., ID179: ‘a community who understood’). A particularly salient idea women wrote of was not feeling ‘alone’ or ‘isolated’ or ‘the only one’ (e.g., ID016: ‘I was so devastated by my diagnosis that I wanted to know I wasn't the only one). Others wrote how being away from judgement was also part of the attraction of such a space (e.g., ID049: ‘to be part of a nonjudgemental likeminded community’). Being linked in with many others who had knowledge and experience of the same condition was highlighted as beneficial, with participants noting group size was an asset in terms of crowdsourcing support: ‘large numbers to get lots of advice’ (ID012), ‘quickest way to access a large number of women’ (ID091) and ‘gain support and information from a large number of women’ (ID105).

#### 3.2.5 Being recommended and self‐referral

Some women focused more specifically on *how* they had joined (*n* = 48), with referral sources typically being people they knew personally or with whom they had interacted with online. Primarily, sources of referral were a friend (*n* = 14), another group/forum online (*n* = 12), family member (*n* = 4), mothers' group (*n* = 4) and healthcare professional (*n* = 3). As one respondent wrote (ID011), she was ‘recommended by a friend who had GD in the past and told me the group was helpful and supportive to her during her GD journey’. With the referring groups online, it was mentioned in a few instances that there were other pregnancy or mothers' groups. Some respondents (*n* = 9) described how they had found the group themselves, and joined through an act of self‐referral.

#### 3.2.6 A safe place to ask questions

The explicit need to ask questions was found in the data (*n* = 22) and thus formed the basis of a category about the desire for a safe ‘place’. One woman (ID217) wrote, ‘to be able to ask questions when educators weren't available’, while another (ID079) reported, ‘if we ever have a question we can ask, no matter what time of day we will always get an answer or be able to talk to someone in the same predicament’; these responses suggested the value of a place to ask questions as an adjunct to formal healthcare. Meanwhile, others indicated they preferred being able to ask questions of their peers: ‘to gain insight other than from a medical professional who sees the issue through a one box fits all approach which isn't realistic’ (ID007), and ‘had questions that I felt safer asking others going through the experience than my medical practitioners’ (ID191). One woman (ID284) wrote that insights from the group were useful for her to ‘ask the correct questions from health care professionals’, indicating that the asking of questions was not contained to the group only but helped fashion her response directly to healthcare providers.

### 3.3 Benefits and values of membership

Perceptions of the value of being in a Facebook group were ascertained via the data collected from the close‐ended question asking what respondents liked about the GD group, and a semantic differential scale which explored perceptions about the community.

#### 3.3.1 Reasons for being in a Facebook group

The two most popular answers as to what was liked about the GD group were ‘reading about food ideas’ (*n* = 277) and ‘finding helpful information and tips’ (*n* = 266), followed by ‘receiving and giving emotional support’ (*n* = 216), ‘being able to visit the group at any time’ (*n* = 215) and ‘discussing my concerns about GD’ (*n* = 208) (see Table 3).

**Table 3:** Responses to close‐ended question (*N* = 340).

Answer	*n*	Response rate %
Reading about food ideas	277	81.5
Finding helpful information and tips	266	78.2
Receiving and giving emotional support	216	63.5
Being able to visit the group at any time	215	63.2
Discussing my concerns about GD	208	61.2
Discussing pregnancy and birth concerns	188	55.3
Seing birth announcements	177	52.1
Other	13	0.04

*Note*: The 13 responses (*n* = 13) in ‘other’ included ‘answers to questions straight away’, ‘personal experience’ and ‘reading previous posts that relate to you’.

Abbreviation: GD, gestational diabetes.

#### 3.3.2 Feelings about the community

**Figure 1:**
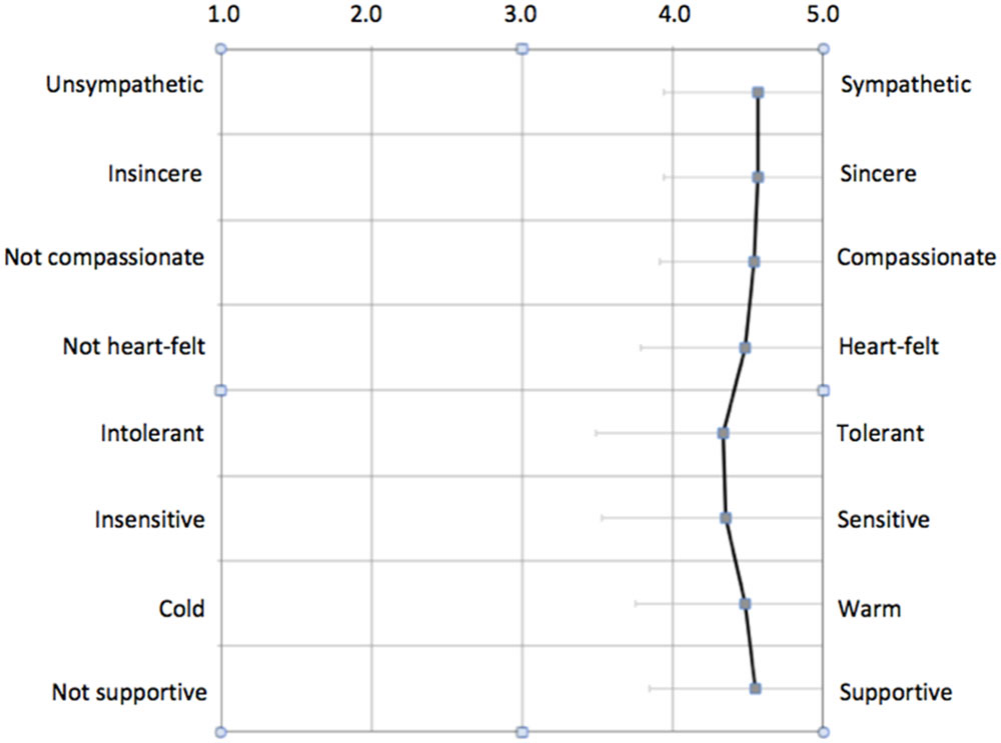
Mean scores and standard deviations across the differential semantic scale.

Results of the semantic differential scale (see Figure 1) suggest that overall, most perceived the community within the Facebook group positively, as sympathetic (*M* = 4.57, SD = 0.63), sincere (*M* = 4.56, SD = 0.62), compassionate (*M* = 4.54, SD = 0.63), heart‐felt (*M* = 4.49, SD = 0.70), tolerant (*M* = 4.33, SD = 0.84), sensitive (*M* = 4.35, SD = 0.82), warm (*M* = 4.48, SD = 0.73) and supportive (*M* = 4.55, SD = 0.71).

## 4 DISCUSSION

Being diagnosed with GDM during pregnancy and its subsequent self‐management can be a challenging experience, and joining a Facebook group is one strategy which some utilise to cope. Although our study focused on Australian groups, we believe these findings are of relevance to many other settings, given the global penetration of Facebook into the lives of ordinary people as well as the rising prevalence of GDM internationally.

Our findings suggest women may have several reasons for seeking out a GDM group, which they join through referral from others as well as self‐referral. Once part of a group, many aspects are valued and, overall, women had a strongly favourable attitude towards such communities. The findings provide depth to this issue while echoing other studies relating to both pregnancy^6^ and diabetes,^21^, ^22^ which found that being in an online group of peers undergoing similar experiences leads to beneficial information and social support. There are differences in the way groups function. In the case of our study, we pooled data from two groups that shared the same overall aims. It was beyond the scope of this study to explore the difference the cultures of these two groups, but we did note some observable differences which may bear some influence on perceptions such as the greater size and volume of posts in one group compared to the other. The evidence from our most recent study, a content analysis of original Facebook posts from across the two groups, did suggest greater intimacy and engagement in the smaller group in relation to emotional help‐seeking posts.^14^

Interestingly, there was more emphasis in the present study on seeking support in the responses to the open‐ended question, whereas the value of information, and food ideas specifically, was emphasised more strongly in the close‐ended question responses. The open‐ended data provided a more nuanced portrait of what support involves in relation to Facebook groups focused on GDM, while the close‐ended data suggests that when respondents wrote about wanting information, this primarily meant food ideas.

Groups such as these ones focused on GDM have been described as ‘communities of practice’ due to the way collective learning occurs through a combination of experiential knowledge and other expert sources.^23^ The experiential knowledge of peers is seen as complementary to medical expertise, sometimes even valued *above* it.^24^ The findings of our study supports this perceived value of ‘experiential knowledge’, as well as building on previous work exploring the value which people place on knowing how others navigate a new diagnosis or health‐related decisions when faced with their own situations, such as chronic conditions including high blood pressure, heart and lung problems and cancer.^25^ Furthermore, the findings of the present study also accord with a recent review^26^ which differentiated *experiential support* as being a feature of such spaces, alongside informational support, emotional support and social support.

It is important to note that the concepts ‘information’ and ‘emotion’ are, in reality, not entirely separate; after all, providing information is a way to address emotional needs—not just for an individual, but for a population. Going further, a study about diabetes communities on Facebook found interactions were not only structured around information and emotion, but for a third purpose as well: community building.^22^ This accords with other findings relating to online peer groups^25^ and is supported by the present study, given respondents clearly expressed desire for community and a sense of belonging. But just as is the case with all communities, there may well be differences in how members appraise group dynamics and experience belongingness. For example, there is currently limited research about the role such communities might play in addressing issues like GDM‐related stigma.^27^ Following these lines of inquiry may yield rich insights into how such groups function and how they specifically support women's self‐management of GDM.

Understandably, healthcare providers may have reservations about the potential spread of misinformation through Facebook groups and lack of professional input; one study of midwives' perceptions, for example, found that midwives recognised women used the internet for pregnancy‐related topics and some worried about misinformation.^28^ A more recent study found midwives did recognise the potential benefits of engaging with families on social media, but felt more training was required for online service delivery and engagement.^29^ As our study demonstrates, Facebook support groups for acute pregnancy‐related conditions such as GDM seem to fill gaps not readily addressed by providers. This includes not only practical advice (e.g., food‐related tips), but also a sense of belonging during a time that can feel particularly isolating.

Given a number of respondents reported that it was a healthcare provider who referred them to the group, clearly some providers do recognise the potential benefits of GDM Facebook groups. This suggests an important avenue of further research, exploring and leveraging Facebook groups, as well as addressing concerns related to providers' understanding of social media‐based support for women with GDM. As formal healthcare cannot be provided ‘no matter what time of day’, such groups can be a valuable adjunct between clinical and self‐management, and even be potentially used to facilitate improved screening for postnatal care given GDM is a marker for increased risk of type 2 diabetes.^30^ These groups are a valuable site for outreach and present a genuine opportunity for formal involvement from healthcare providers. As our study indicates, these groups are also underutilised as a means for accessing and involving individuals in research. Although we examined Facebook groups in particular given Facebook is the most utilised social media platform globally, with the continued growth and utilisation of its group function, the popularity of other social media (e.g. Instagram, TikTok, Reddit) certainly warrants further investigation in relation to GDM. Future research can inform clinical practice, as well as ensure clinicians keep abreast of developments in how women are self‐managing based on what they are exposed to online.

A significant strength of the study is the very large proportion of respondents who answered the open‐ended question. This is far from typical; the nonresponse rate is generally much higher for open‐ended questions than for closed‐ended questions in web surveys.^31^ Data from open‐ended questions are open to interpretation as to how they are answered and do not necessarily provide rich data but, as noted elsewhere, if the question asked is specific—as in the present study—the results can be invaluable, especially when considered together with quantitative findings.^32^ A key limitation of this study is that the sample examined was self‐selected, and survey respondents were more highly‐educated than the general population, largely Australian‐born and, for the most part, culturally homogenous. Thus, findings are not necessarily generalisable to other populations, even within Australia. These demographics point to key gaps to address in the future to ensure a more representative sample, given current research suggest a potentially higher at‐risk profile for GDM for women who migrate^33^ and from certain ethnicities,^34^ though the international evidence is still inconclusive on these being risk factors.

## 5 CONCLUSION

Our study examined motivations to join GDM online support groups and the perceived benefits of membership. Findings suggest that such groups are valuable spaces that provide women with access to tailored emotional and informational support for GDM, including access to embodied, practical, crowdsourced information; the ability to ask questions; and belonging to a national community of peers. GDM Facebook groups can enhance women's emotional wellbeing and fill gaps not addressed by healthcare providers.

## AUTHOR CONTRIBUTIONS

**Sheila Pham**: Writing—original draft; writing—review and editing; conceptualisation; investigation; methodology; data curation; formal analysis. **Kate Churruca**: Writing—review and editing; conceptualisation; methodology; supervision; formal analysis. **Louise A. Ellis**: Writing—review and editing; conceptualisation; supervision; formal analysis. **Jeffrey Braithwaite**: Writing—review and editing; conceptualisation; funding acquisition; supervision.

## CONFLICT OF INTEREST STATEMENT

The authors declare no conflict of interest.

## ETHICS STATEMENT

Prior to the commencement of data collection, S. P. requested permission from the administrators of both groups to join to conduct research, and disclosed her positionality as someone who had experienced GDM. Ethics approval for the study was gained from Macquarie University's Human Research Ethics Committee (Reference: 5201827734364, date of approval 27 May 2018). Participants provided voluntary informed consent and all survey responses were anonymous.

## Supporting information

Supporting information.

## Data Availability

Research data are not shared.
