# Supplementary material for: ‘No matter what time of day’: The value of joining Facebook groups supporting women's self‐management of gestational diabetes mellitus
Source: Health Expect. 2024 May 24;27(3):e14082. doi: 10.1111/hex.14082 (PMC11116941; doi:10.1111/hex.14082)
Supplement: Supplementary file 1 — Supporting information. [file HEX-27-e14082-s001.docx]

**Appendix 1. Survey questions presented in the study**

1. Can you explain exactly what led you to join a GD Facebook group?

2. What do you like about being in a GD group? Please select all that apply.

☐ Discussing my concerns about GD

☐ Reading about food ideas

☐ Discussing pregnancy and birth concerns

☐ Seeing birth announcements

☐ Finding helpful information and tips

☐ Being able to visit the group at any time

☐ Receiving and giving emotional support

☐ Other (please specify):

3. During my interactions in a GD Facebook group, I felt the community to be:

Unsympathetic o o o o o Sympathetic

Insincere o o o o o Sincere

Not compassionate o o o o o Compassionate

Not heart-felt o o o o o Heart-felt

Intolerant o o o o o Tolerant

Insensitive o o o o o Sensitive

Cold o o o o o Warm

Not supportive o o o o o Supportive

**Demographic questions**

1. What is your age (in years)?

2. In which country were you born?

- Australia
- Other (please specify):

3. Do you speak a language other than English at home?

4. Do you have a family history of diabetes, including GD?

5. What is the highest level of education that you have completed? If you are currently studying, but have not completed a qualification, please answer with the highest level you have completed.

- Less than year 12
- Year 12 (HSC) or equivalent
- TAFE qualification, technical, trade certificate, diploma or equivalent
- Bachelors degree
- Postgraduate degree or higher
- Don't know
